# Development and Initial Validation of a Brief Questionnaire on the Patients’ View of the In-Session Realization of the Six Core Components of Acceptance and Commitment Therapy

**DOI:** 10.32872/cpe.v2i3.3115

**Published:** 2020-09-30

**Authors:** Thomas Probst, Andreas Mühlberger, Johannes Kühner, Georg H. Eifert, Christoph Pieh, Timo Hackbarth, Johannes Mander

**Affiliations:** a Department for Psychotherapy and Biopsychosocial Health, Danube University Krems, Krems, Austria; b Department of Psychology, Regensburg University, Regensburg, Germany; c Practice for Psychotherapy, Würzburg, Germany; d Department of Psychology, Chapman University, Orange, CA, USA; e Center for Psychological Psychotherapy, Heidelberg University, Heidelberg, Germany; Philipps-University of Marburg, Marburg, Germany

**Keywords:** Acceptance and Commitment Therapy, session report, reliability, validity

## Abstract

**Background:**

Assessing in-session processes is important in psychotherapy research. The aim of the present study was to create and evaluate a short questionnaire capturing the patients’ view of the in-session realization of the six core components of Acceptance and Commitment Therapy (ACT).

**Method:**

In two studies, psychotherapy patients receiving ACT (Study 1: n = 87) or Cognitive-Behavioral Therapy (CBT) (Study 2, Sample 1: n = 115; Sample 2: n = 156) completed the ACT session questionnaire (ACT-SQ). Therapists were n = 9 ACT therapists (Study 1) and n = 77 CBT trainee therapists (Study 2).

**Results:**

Factor structure: Exploratory factor analyses suggested a one-factor solution for the ACT-SQ. Reliability: Cronbach’s alpha of the ACT-SQ was good (Study 1: α = .81; Study 2, Sample 1: α = .84; Sample 2: α = .88). Convergent validity: The ACT-SQ was positively correlated with validated psychotherapeutic change mechanisms (p < .05). Criterion validity: Higher ACT-SQ scores were associated with better treatment outcomes (p < .05).

**Conclusion:**

The study provides preliminary evidence for the reliability and validity of the ACT-SQ to assess the in-session realization of the six core components of ACT in the patients’ view. Further validation studies and ACT-SQ versions for therapists and observers are necessary.

## Abstract (highlights)

- The ACT-SQ is a patient self-report on the in-session realization of the six core components of ACT.
- Data of two studies (ACT, CBT therapies) support the reliability and validity of the ACT-SQ.
- Further validation studies and ACT-SQ versions for therapists and observers are necessary.

Acceptance and Commitment Therapy (ACT; Hayes, 2004) is one of the third-wave cognitive-behavioral therapies (CBT). Several reviews and meta-analyses summarized the effectiveness of ACT for various clinically relevant problems (A-Tjak et al., 2015; Graham, Gouick, Krahé, & Gillanders, 2016; Öst, 2014; Powers, Zum Vörde Sive Vörding, & Emmelkamp, 2009; Swain, Hancock, Hainsworth, & Bowman, 2013). A central treatment strategy in ACT is reducing the patients’ psychological inflexibility and thereby increasing psychological flexibility. The ACT model of psychological flexibility consists of the following of six core components (see Table 1): acceptance, cognitive defusion, contact with the present moment, self-as-context, values, and committed action. These six core components of psychological flexibility can be described as mindfulness and acceptance processes (acceptance, cognitive defusion, contact with the present moment, self-as-context) as well as commitment and behavior change processes (contact with the present moment, self-as-context, values, and committed action). The counterparts of these six components of psychological flexibility are formulated in the ACT model of psychological inflexibility (see Table 1): experiential avoidance (vs. acceptance), cognitive fusion (vs. cognitive defusion), dominance of the conceptualized past and feared future (vs. contact with the present moment), attachment to the conceptualized self (vs. self-as-context), lack of values (vs. values), and inaction, impulsivity, or avoidant persistence (vs. committed action).

**Table 1:** ACT Model of Psychological Flexibility and ACT Model of Psychological Inflexibility

ACT model of psychological flexibility		ACT model of psychological inflexibility	
*Component*	Description	*Component*	Description
*Acceptance*	Being open towards all experiences	*Experiential avoidance*	Avoiding unwanted experiences
*Cognitive defusion*	Observing thoughts and inner experiences come and go	*Cognitive fusion*	Being entangled in one’s thoughts and inner experiences
*Contact with the present moment*	Non-judgmental awareness of current experiences	*Dominance of the conceptualized past and feared future*	Ruminating on the past or worrying about the future
*Self-as-context*	Being aware of one’s experiences without attachment to them	*Attachment to the conceptualized self*	Inflexible identification with a self-image
*Values*	Having identified valued directions	*Lack of values*	Having no orientation in life
*Committed action*	Effective behavior related to one’s values	*Inaction, impulsivity, or avoidant persistence*	Problems to keep either commitments or to set goals

A meta-analysis on laboratory-based component studies revealed positive effects for treatment strategies on the six ACT core components (Levin, Hildebrandt, Lillis, & Hayes, 2012). Moreover, psychotherapy research has shown that patients who improve their skills in acceptance, cognitive defusion, contact with the present moment, and values-based actions during therapy show better treatment outcomes (e. g., Åkerblom, Perrin, Rivano Fischer, & McCracken, 2015; Arch, Wolitzky-Taylor, Eifert, & Craske, 2012b; Baranoff, Hanrahan, Kapur, & Connor, 2013; Forman, Herbert, Moitra, Yeomans, & Geller, 2007; Forman et al., 2012; Hesser, Westin, & Andersson, 2014; Niles et al., 2014; Vowles & McCracken, 2008; Zettle, Rains, & Hayes, 2011). Interestingly, some of these studies found improvements in ACT processes to be beneficial for the outcome not only in ACT but also in CBT as well as multidisciplinary treatments. ACT processes might therefore be change mechanisms in other psychotherapies than ACT as well, i. e. general change mechanisms. Some ACT processes were even more strongly associated with the outcome in CBT than in ACT, for example in the study by Arch et al. (2012b) in which cognitive defusion predicted worry reductions more in CBT than in ACT.

Several questionnaires have been published to measure a patient’s skill in the ACT components: e. g., Acceptance and Action Questionnaire II (Bond et al., 2011); Acceptance and Action Questionnaire for University Students (Levin, Krafft, Pistorello, & Seeley, 2019); Comprehensive assessment of Acceptance and Commitment Therapy processes (Francis, Dawson, & Golijani-Moghaddam, 2016); Chronic Pain Acceptance Questionnaire (McCracken, Vowles, & Eccleston, 2004), Cognitive Fusion Questionnaire (Gillanders et al., 2014), Multidimensional Experiential Avoidance Questionnaire (Gámez, Chmielewski, Kotov, Ruggero, Suzuki, & Watson, 2014), Tinnitus Acceptance Questionnaire (Weise, Kleinstäuber, Hesser, Westin, & Andersson, 2013), The Valued Living Questionnaire (Wilson, Sandoz, Kitchens, & Roberts, 2010). How strong patients improve their skills in ACT components might depend on the in-session realization of the ACT components. As far as we know, no study has yet explored this research question. This might be because only the observer-based Drexel University ACT/CBT Therapist Adherence Rating Scale (DUTARS; McGrath, 2012) is available to measure the degree the ACT components are realized in a psychotherapy session. The DUTARS was applied in previous clinical trials on ACT to assess treatment adherence (Arch et al., 2012a; Gloster et al., 2015). Although such observer-based measures provide valuable data, there are several barriers to apply observer-based ratings in psychotherapy, especially under the conditions of routine practice. For example, observers must be trained to provide reliable and valid data, financial or other compensations are necessary since observing sessions or session segments consumes a serious amount of time (Weck, Grikscheit, Höfling, & Stangier, 2014), and only certain consent to being observed in-session limiting the generalizability of the results.

Besides observer ratings, ratings given by patients are complementary data sources. Patient ratings on in-session processes are easier to obtain than observer ratings. Patients can fill out session questionnaires directly after the psychotherapy session to measure the degree therapeutic factors were realized in this given psychotherapy session. Patient ratings of in-session processes are especially relevant as they correlate most consistently with psychotherapy outcome (e. g, Horvath & Symonds, 1991; Mander et al., 2013, 2015; Ogrodniczuk, Piper, Joyce, & McCallum, 2000). Several session questionnaires were published on the in-session realization of the therapeutic alliance (Horvath & Greenberg, 1989) and the psychotherapeutic change processes according to Grawe (1997): problem actuation (activation of problems and related emotions), clarification of meaning (acquiring new insights and a deeper understanding of the problems), resource activation (recognizing potential, strengths, and positive facets), and mastery (the ability to cope with problems) (see Mander et al., 2013, 2015). Yet, no session report exists, to our knowledge, which captures the in-session realization of the six core components of ACT. A brief, time-economic and psychometrically sound ACT session report would have the potential to enrich psychotherapy research as well as clinical practice. Clinical implications would be that this measure could be applied in more settings than the observer-based DUTARS and that therapists could use this measure to obtain feedback on the patients’ perspective of the in-session realization of the ACT components.

In the present study, we developed and evaluated a brief ACT session questionnaire (ACT-SQ; see Supplementary Materials). The ACT-SQ was created to obtain patient ratings on the in-session realization of the ACT components of psychological flexibility. In this manuscript, we present two studies. Study 1 investigated the factor structure, the reliability, and the convergent validity. Study 2 analyzed the factor structure, the reliability, the convergent validity, and also criterion validity. The following research questions were evaluated:

1. What is the factor structure of the ACT-SQ?
2. How is the reliability (internal consistency) of the ACT-SQ?
3. With regard to convergent validity: How are the associations between the ACT-SQ and general change mechanisms? The general change mechanisms proposed by Grawe (1997) – problem actuation, clarification of meaning, resource activation, mastery – were used to evaluate convergent validity. The general change mechanisms of Grawe were used to test convergent validity due to two reasons. First, these general change mechanisms are considered to be relevant in all psychotherapies, therefore also in ACT. Second, ACT processes might also be general psychotherapeutic change mechanisms, since – as mentioned above – improvements in ACT processes have been found to beneficial for the outcome not only in ACT but also in CBT and multidisciplinary treatments.
4. Are the factor structure, reliability, and convergent validity of the ACT-SQ comparable between a sample of patients treated with ACT (Study 1) and a sample of patients treated with CBT (Study 2)? ACT and CBT have similarities and differences (Arch & Craske, 2008; Harley, 2015) so that the factor structure, reliability, and convergent validity of the ACT-SQ might resemble more the similarities or the differences.
5. Regarding criterion validity: Is the ACT-SQ associated with treatment outcomes?
6. Are the factor structure, reliability, convergent validity, and criterion validity of the ACT-SQ comparable in different treatment phases? It has been discussed that the earlier and later phases of psychotherapy differ for example in common factors (Ilardi & Craighead, 1994; Lambert, 2005) so that the factor structure, reliability, convergent validity, and criterion validity of the ACT-SQ might depend on the treatment phase.

## Study 1

### Method

The study was performed according to the resolution of Helsinki and the professional obligations for therapists. No ethics committee was involved in Study 1 because no harmful procedures were applied and questionnaire-data were collected anonymously. The responsible psychotherapists asked their patients to take part in the study. The informed consent of the participants was implied through questionnaire completion. The anonymized questionnaires were sent by the therapists to the first author.

#### Measures

The following two questionnaires were administered simultaneously to the patients during psychotherapy: the newly developed ACT-SQ and the psychometrically sound patient version of the “Scale for the Multiperspective Assessment of General Change Mechanisms in Psychotherapy” (SACiP; Mander et al., 2013).

The SACiP evaluates the degree the therapeutic alliance and other change mechanisms according to Grawe (1997) were realized in the given psychotherapy session. The SACiP consists of adapted items from the German shortened version of the Working Alliance Inventory (WAI-S; Munder, Wilmers, Leonhart, Linster, & Barth, 2010) as well as from the Bernese Post Session Report (BPSR; Flückiger et al., 2010). Factor analyses revealed the following six SACiP scales: emotional bond, agreement on collaboration, problem actuation, clarification of meaning, mastery, and resource activation (Mander et al., 2013). The emotional bond scale and the agreement on collaboration scale measure aspects of the therapeutic alliance, the problem actuation scale assesses how strong problems as well as related emotions were activated in the session, the clarification of meaning scale measures the new insights the patient gained into his/her behavior during the session, the mastery scale assesses the degree the session helped the patients to cope with his/her problems, and the resource activation scale measures how strong the patients’ strengths were used in-session. The measure demonstrated an excellent factor structure with factor loadings of .51 ≤ λ ≤ .85. Confirmatory factor analyses supported the exploratory model. The instrument revealed good to excellent internal consistencies with .71 ≤ α ≤ .90. Studies also demonstrated criterion validity since treatment outcome was significantly predicted by all change mechanisms except for problem actuation (e.g. Mander et al., 2013, 2015). Example items of the SACiP patient version are the following: “Today, I felt comfortable in the relationship with the therapist” (emotional bond), “In today’s session, I was highly emotionally involved” (problem actuation), “Today, the therapist intentionally used my abilities for therapy” (resource activation), “Today, I became more aware of the motives for my behavior” (clarification of meaning), “Today, the therapist and I worked toward mutually agreed upon goals” (agreement on collaboration), “Today, we really made progress in therapy in overcoming my problems (mastery).

In the ACT-SQ, patients rate how strong the ACT components of psychological flexibility were realized in psychotherapy sessions on a five point Likert scale. Each item of the ACT-SQ represents one ACT component. Six pilot items of the ACT-SQ were formulated by T.P. on the basis of the ACT literature. T.P. then discussed the items with CBT psychotherapists with ACT expertise (J.K., G.H.E., and A.M.). The experts gave feedback regarding the fit of the items to the ACT model and provided concrete suggestions how the items could be optimized. The six pilot items were changed and refined accordingly. The resulting six items represent the items of the final ACT-SQ and were used in the present study (the ACT-SQ is available license free, the German and English version are included in the Appendix, see Supplementary Materials).

#### Participants

Therapists: The *n* = 69 ACT therapists listed in the German section of the Association for Contextual Behavioral Science (Deutschsprachige Gesellschaft für kontextuelle Verhaltenswissenschaften e.V.; DGKV) were invited to participate in October 2015 and the *n* = 68 ACT therapists listed in the e-mail list of the German ACT network were invited to partake in December 2014. Therefore, therapists listed in both the German section of the Association for Contextual Behavioral Science and the e-mail list of the German ACT network were contacted twice. Nine ACT therapists (see Acknowledgements) took part and encouraged their patients to fill in the ACT-SQ and the SACiP after one psychotherapy session. The nine ACT therapists were certified in cognitive-behavioral therapy (CBT) and their average work experience with ACT amounted to *M* = 4.56 years (*SD* = 2.46).

Patients: Eighty-seven patients treated by the *n* = 9 ACT therapists completed the ACT-SQ after the *M* = 21.25^th^ psychotherapy session (*SD* = 19.84). The description of the participating *N* = 87 patients is given in Table 2. The diagnoses were made by the responsible therapist.

**Table 2:** Description of the Patients of Study 1

Gender	*n*	%
male	33	37.9
female	53	60.9
no data	1	1.2
Diagnoses according to chapter V of the ICD-10 (all diagnoses, not only primary diagnosis)	***n***	**%**
F4	53	40.2
F3	46	34.8
F1	15	11.4
F6	8	6.1
others	10	7.6
Outpatients / Inpatients	*n*	%
outpatient	78	89.7
inpatient	9	10.3
Comorbidity: Amount of diagnoses according to chapter V of the ICD-10	*M*	*SD*
	1.54	0.71
Age at time of assessment	*M*	*SD*
	42.48	14.79

*Note.* F4 = Neurotic, stress-related and somatoform disorders; F3 = Mood (affective) disorders; F1 = Mental and behavioural disorders due to psychoactive substance use; F6 = Disorders of adult personality and behavior. Number of diagnoses higher than number of patients since multiple diagnoses per patients are possible.

#### Analyses

SPSS 25 was used to perform the statistical analyses. Means (*M*), standard deviations (*SD*), frequencies (*n*), and percentages (%) were calculated for the sample description. To explore the factor structure of the ACT-SQ, an exploratory factor analysis (EFA) with maximum likelihood estimation and with oblique rotation (oblimin direct) was performed. The Kaiser criterion (factors with eigenvalues larger than 1 were retained), the Kaiser-Meyer-Olkin Measure of Sampling Adequacy (KMO), and the Bartlett’s Test of Sphericity were applied. Cronbach’s alpha (α) was computed to measure reliability. Furthermore, Pearson correlation coefficients (*r*) were calculated to measure correlations between the ACT-SQ and general change mechanisms (convergent validity). All statistical tests were performed two-tailed and the significance value was set to *p* < .05. Results will be presented with and without Bonferroni-correction for multiple comparisons.

### Results

Factor structure and reliability: The EFA produced a KMO value of .79 and the Bartlett’s test reached significance, χ^2^(15) = 150.04; *p* < .01. The eigenvalues amounted to 3.06, 0.85, 0.73, 0.58, 0.44, 0.34. Therefore, only one factor was retained when Kaiser’s criterion was applied. The loadings of the six items are presented in Table 3. There were no cross-loadings. Cronbach’s alpha (α) across all six items amounted to α = .81.

**Table 3:** Loadings of the ACT-SQ in Study 1

The last (XY) psychotherapy session(s) helped me…	Loading λ
Item 1 Acceptance “…to accept unpleasant feelings, thoughts or body sensations rather than fight them”	.58
Item 2 Cognitive defusion “…to gain more inner distance from unpleasant feelings, thoughts or body sensations and to observe them rather than getting caught up in them”	.65
Item 3 Contact with the present moment “…to stay in the here and now (in the present moment) rather than concerning myself with my future and my past”	.60
Item 4 Self-as-context “…to realize that my feelings, thoughts and body sensations are part of me, but that I am more than my feelings, thoughts and body sensations”	.72
Item 5 Values “…to recognize what is important to me in my life and what gives orientation to my life”	.61
Item 6 Committed action “…to act in daily life according to what is important to me in my life and what gives orientation to my life”	.70

*Note.* Sample of Study 1: *N* = 87 patients treated by *n* = 9 ACT therapists.

Correlations with general change mechanisms: The associations between the ACT-SQ mean score and the mean scores of the SACiP scales are presented in Table 4. Before applying Bonferroni correction (*p* < .05), the ACT-SQ was significantly correlated with all general change mechanisms except for problem actuation. The association between the ACT-SQ and the emotional bond, however, was not significant anymore after (*p* < .008) applying Bonferroni correction (*p* = .05 / 6 comparisons).

**Table 4:** Correlations Between the ACT-SQ and the SACiP Scales in Study 1

Variable	SACiP					
	Emotional bond	Problem actuation	Resource activation	Clarification of meaning	Agreement on collaboration	Mastery
**ACT-SQ**	.23*	.10	.55**	.43**	.40**	.64**

*Note.* Sample of Study 1: *N* = 87 patients treated by *n* = 9 ACT therapists. ACT-SQ = ACT Session Questionnaire; SACiP = Scale for the Multiperspective Assessment of General Change Mechanisms in Psychotherapy.

*p < .05. **p < .001.

### Discussion

The results provide preliminary evidence for the factor structure, the reliability, and the convergent validity of the ACT-SQ. Regarding Research Question 1, we found a one-factor solution. Results for Research Question 2 indicate a good reliability. Convergent validity (Research Question 3) was supported by significant correlations between the ACT-SQ and general change mechanisms except for problem actuation. A limitation of the study is the relatively small sample size of participating ACT therapists. Future research could use recently published recommendations on how to motivate therapists for psychotherapy research (Taubner, Klasen, & Munder, 2016) to obtain larger samples. Moreover, no associations between the ACT-SQ and treatment outcomes (criterion validity) were evaluated. Therefore, Study 2 was planned to investigate the criterion validity of the ACT-SQ. Another aim was to investigate whether the factor structure, the reliability, and the convergent validity as shown in Study 1 can be replicated in Study 2.

## Study 2

### Method

The methods of Study 2 were approved by the local ethics committee (Ethikkommission der Fakultät für Verhaltens- und Empirische Kulturwissenschaften der Universität Heidelberg) and written informed consent was obtained from the patients.

#### Measures

The ACT-SQ and the SACiP (see measures in Study 1) were administered to patients after the 15^th^ therapy session and at the end of psychotherapy. Furthermore, the German versions of the Brief Symptom Inventory (BSI; Franke, 2000) and the Beck Depression Inventory (BDI-II; Hautzinger, Keller, & Kühner, 2009) were administered as outcome measures at pre-treatment and post-treatment as well as after the 15^th^ psychotherapy session. The Global Severity Index (GSI) of the BSI and the total score of the BDI-II were used in the study at hand. These measures are reliable and valid (see for example, Franke, 2000 for the German version of BSI; Derogatis & Melisaratos, 1983 for the English version of BSI; Kühner et al., 2007 for the German version of BDI-II; Beck & Steer, 1998 for the English version of BDI-II). references. Cronbach’s alpha (α) values have been reported to be high: between .92 and .96 for the GSI of the German BSI and ≥ .84 for the German BDI-II.

#### Participants

**Figure 1:**
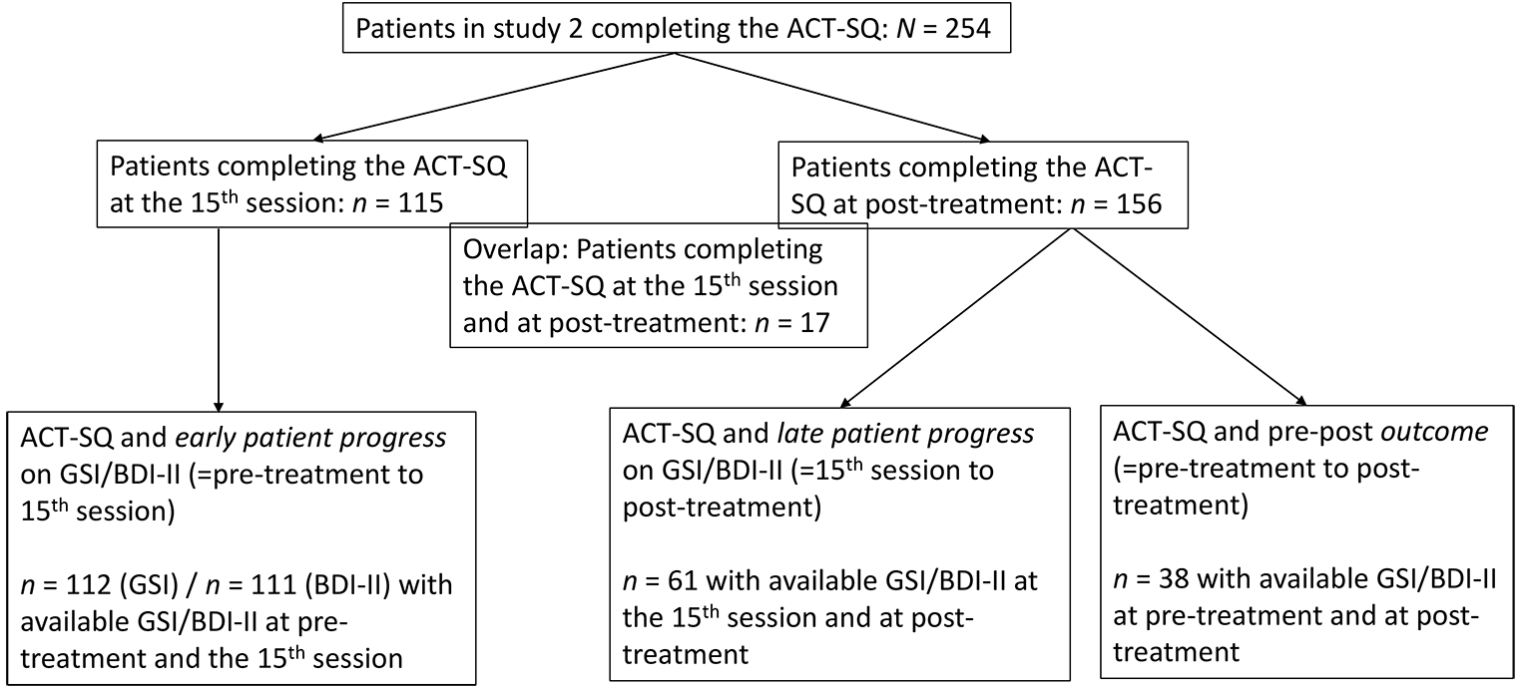
Flow-Chart

Therapists and patients were different from the therapists and patients included in Study 1. Between November 2016 and November 2017, *n* = 77 CBT trainee therapists working at a large outpatient training center took part. These therapists treated the *n* = 254 patients who completed the ACT-SQ: *n* = 115 outpatients completed the ACT-SQ after the 15^th^ CBT session and *n* = 156 outpatients completing the ACT-SQ at the end of CBT (post-treatment). As the ACT-SQ was implemented for ongoing and new therapies, these two patient sample were independent from each other except for *n* = 17 patients who completed the ACT-SQ at both assessment points. A subset of patients filling in the ACT-SQ also provided data for the outcome measures (see flow-chart in Figure 1) and their data was used to evaluate associations between the ACT-SQ and pre-post outcome as well as early and late patient progress (Research Questions 5 and 6).

The patients answering the ACT-SQ at the end of CBT had on average *M* = 39.68 (*SD* = 14.98) individual therapy sessions. The description of the participating patients is given in Table 5. Structured clinical interviews (SCID) were used to make the diagnoses.

**Table 5:** Description of the Patients of Study 2

Variable	15^th^ session sample		Post-treatment sample	
	*n*	%	*n*	%
Gender				
male	51	44.3	68	43.6
female	64	55.7	88	56.4
Diagnoses according to chapter V of the ICD-10 (all diagnoses, not only primary diagnosis)				
F4	68	36.0	87	34.3
F3	72	38.1	102	40.2
F1	10	5.3	16	6.3
F6	22	11.6	23	9.1
others	17	9.0	26	10.2
	*M*	*SD*	*M*	*SD*
Comorbidity: Amount of diagnoses according to chapter V of the ICD-10				
	1.64	.84	1.63	.87
Age at time of assessment				
	36.50	13.03	35.73	13.60

*Note.* F4 = Neurotic, stress-related and somatoform disorders; F3 = Mood (affective) disorders; F1 = Mental and behavioural disorders due to psychoactive substance use; F6 = Disorders of adult personality and behavior. Number of diagnoses higher than number of patients since multiple diagnoses per patients are possible.

#### Analyses

SPSS 25 was used to perform the statistical analyses. Means (*M*), standard deviations (*SD*), frequencies (*n*), and percentages (%) were calculated for the sample description. An EFA with maximum likelihood estimation and oblique rotation (oblimin direct) was performed to investigate the factor structure of the ACT-SQ. The Kaiser criterion (factors with eigenvalues larger than 1 were retained), the Kaiser-Meyer-Olkin Measure of Sampling Adequacy (KMO), and the Bartlett’s Test of Sphericity were applied. Cronbach’s alpha (α) was computed to measure reliability. Furthermore, Pearson correlation coefficients (*r*) were calculated to measure associations between the ACT-SQ and general change mechanisms (convergent validity). Moreover, associations between the ACT-SQ and treatment outcome were explored with linear regression analyses. To measure the pre-post outcome, the outcome measure (GSI, BDI-II) at post-treatment was the dependent variable and the ACT-SQ at post-treatment as well as the outcome measure (GSI, BDI-II) at pre-treatment were independent variables. We also investigated associations between the ACT-SQ and early as well as late patient progress. For early patient progress, the patient reported outcome measure (GSI, BDI-II) at the 15^th^ CBT session was the dependent variable and the ACT-SQ at the 15^th^ CBT session as well as the outcome measure (GSI, BDI-II) at pre-treatment were independent variables. For late patient progress, the patient reported outcome measure (GSI, BDI-II) at post-treatment was the dependent variable and the ACT-SQ at post-treatment as well as the outcome measure (GSI, BDI-II) at the 15^th^ CBT session were independent variables. We also performed these analyses without the ACT-SQ as independent variable to evaluate how the R2-squared values change when including the ACT-SQ as independent variable. All statistical tests were performed two-tailed and the significance value was set to *p* < .05. Results will be given with and without Bonferroni-correction for multiple comparisons.

### Results

Factor structure and reliability for the 15^th^ CBT session sample: The EFA produced a KMO value of .86 and the Bartlett’s test was significant, χ^2^(15) = 235.14; *p* < .01. The eigenvalues were 3.33, 0.81, 0.54, 0.50, 0.46, 0.36. Only one factor was retained when Kaiser’s criterion was applied. The loadings of the six items are given in Table 6. There were no cross-loadings. Cronbach’s alpha (α) across all six items was α = .84 for the 15^th^ CBT session sample.

**Table 6:** Loadings of the ACT-SQ in Study 2

The last (XY) psychotherapy session(s) helped me…	Loading λ	
	15^th^ session sample	Post-treatment sample
Item 1 Acceptance “…to accept unpleasant feelings, thoughts or body sensations rather than fight them”	.53	.66
Item 2 Cognitive defusion “…to gain more inner distance from unpleasant feelings, thoughts or body sensations and to observe them rather than getting caught up in them”	.78	.73
Item 3 Contact with the present moment “…to stay in the here and now (in the present moment) rather than concerning myself with my future and my past”	.65	.78
Item 4 Self-as-context “…to realize that my feelings, thoughts and body sensations are part of me, but that I am more than my feelings, thoughts and body sensations”	.67	.69
Item 5 Values “…to recognize what is important to me in my life and what gives orientation to my life”	.67	.84
Item 6 Committed action “…to act in daily life according to what is important to me in my life and what gives orientation to my life”	.78	.79

*Note.* 15^th^ session sample of Study 2: *n* = 115 patients; post-treatment sample of Study 2: *n* = 156 patients; both samples treated by *n* = 77 CBT trainee therapists.

Factor structure and reliability for the post-treatment sample: For the EFA, the KMO value was .87 and the Bartlett’s test reached significance, χ^2^(15) = 450.37; *p* < .01. The eigenvalues were 3.79, 0.58, 0.54, 0.44, 0.40, 0.25. Only one factor was retained when Kaiser’s criterion was applied. The loadings of the six items are shown in Table 6. There were no cross-loadings. Cronbach’s alpha (α) across all six items amounted to α = .88 for the CBT post-treatment sample.

Correlations with general change mechanisms: The associations between the ACT-SQ mean score and the mean scores of the SACiP scales at CBT session 15^th^ and at post-treatment are shown in Table 7. The correlations were all positive and statistically significant before (*p* < .05) and after (*p* < .004) correcting for multiple testing (*p* = .05 / 12 comparisons).

**Table 7:** Correlations Between the ACT-SQ and the SACiP Scales in Study 2

ACT-SQ	SACiP					
	Emotional bond	Problem actuation	Resource activation	Clarification of meaning	Agreement on collaboration	Mastery
15^th^ session sample	.40**	.42**	.75**	.73**	.54**	.78**
Post-treatment sample	.49**	.59**	.78**	.74**	.66**	.83**

*Note.* 15^th^ session sample of Study 2: *n* = 115 patients; post-treatment sample of Study 2: *n* = 156 patients; both samples treated by *n* = 77 CBT trainee therapists. ACT-SQ = ACT Session Questionnaire; SACiP = Scale for the Multiperspective Assessment of General Change Mechanisms in Psychotherapy.

**p < .001.

Associations with treatment outcome: The results of the linear regression models are summarized in Table 8. The results indicate that higher ACT-SQ scores were associated with more beneficial pre-post outcome as well as with early and late patient progress before (*p* < .05) and after (*p* < .008) Bonferroni correction (*p* = .05 / 6 comparisons).

**Table 8:** Associations Between the ACT-SQ and Treatment Outcomes

Dependent variable / Parameter	Unstandardized coefficient B		Standardized Coefficient β	*t*	*p*
	β	*SE*			
Outcome					
GSI at post-treatment (*n* = 38)					
Constant	1.19	0.23		5.13	< .001
GSI at pre-treatment	0.40	0.10	0.47	3.97	< .001
ACT-SQ at post-treatment	-0.36	0.07	-0.59	-5.00	< .001
BDI-II at post-treatment (*n* = 38)					
Constant	25.56	3.91		6.55	< .001
BDI-II at pre-treatment	0.33	0.09	0.34	3.85	< .001
ACT-SQ at post-treatment	-7.91	0.99	-0.71	-8.03	< .001
Early patient progress					
GSI at 15^th^ therapy session (*n* = 112)					
Constant	0.66	0.17		3.83	< .001
GSI at pre-treatment	0.70	0.06	0.72	11.78	< .001
ACT-SQ at 15^th^ therapy session	-0.20	0.06	-0.21	-3.36	.001
BDI-II at 15^th^ therapy session (*n* = 111)					
Constant	13.86	3.05		4.54	< .001
BDI-II at pre-treatment	0.62	0.06	0.65	9.96	< .001
ACT-SQ at 15^th^ therapy session	-4.42	1.02	-0.28	-4.31	< .001
Late patient progress					
GSI at post-treatment therapy session (*n* = 61)					
Constant	0.79	0.22		3.54	.001
GSI at 15^th^ therapy session	0.63	0.11	0.53	5.72	< .001
ACT-SQ at post-treatment	-0.25	0.06	-0.38	-4.09	< .001
BDI-II at post-treatment session (*n* = 61)					
Constant	18.65	4.06		4.59	< .001
BDI-II at 15^th^ therapy session	0.51	0.10	0.45	5.04	< .001
ACT-SQ at post-treatment	-5.77	1.06	-0.49	-5.45	< .001

*Note. SE* = Standard Error; ACT-SQ = ACT Session Questionnaire; GSI = Global Severity Index of the Brief Symptom Inventory; BDI-II = Beck Depression Inventory.

For the pre-post outcome, the *R*-squared values were .17 (GSI) and .28 (BDI-II) when predicting the outcome measure at post-treatment by the outcome measure at pre-treatment and the *R*-squared values changed to .52 (GSI) and .75 (BDI-II) when predicting the outcome measure at post-treatment by the outcome measure at pre-treatment as well as by the ACT-SQ.

For the early patient progress, the R-squared values were .56 (GSI) and .46 (BDI-II) when predicting the outcome measure at the 15^th^ session by the outcome measure at pre-treatment and the R-squared values changed to .60 (GSI) and .54 (BDI-II) when predicting the outcome measure at the 15^th^ session by the outcome measure at pre-treatment as well as by the ACT-SQ.

For late patient progress, the R-squared values were .44 (GSI) and .49 (BDI-II) when predicting the outcome measure at post-treatment by the outcome measure at the 15^th^ session and the R-squared values changed to .57 (GSI) and .67 (BDI-II) when predicting the outcome measure at post-treatment by the outcome measure at the 15^th^ session as well as by the ACT-SQ.

### Discussion

Study 2 supported the one-factor solution (Research Question 1), a good reliability (Research Question 2), as well as associations between the ACT-SQ and general change mechanisms (convergent validity, Research Question 3). The results were comparable to the results obtained in Study 1 with the exception that the general change mechanism problem actuation was correlated with the ACT-SQ only in Study 2 (Research Question 4). The results indicate that the ACT-SQ has many similarities in ACT and CBT but that there are also differences (Research Question 5): the overlap between the in-session realization of problem actuation and the ACT components was specific for CBT. Criterion validity was not evaluated in Study 1 (ACT) but the significant associations between the ACT-SQ and pre-post outcome in Study 2 (CBT) indicate criterion validity (Research Question 5). Despite possible differences between earlier and later treatment phases (Ilardi & Craighead, 1994; Lambert, 2005), the factor structure, reliability, convergent validity, and criterion validity of the ACT-SQ were comparable in the earlier and later treatment phases (Research Question 6). A limitation of Study 2 is that the sample size on associations between the ACT-SQ and pre-post outcome was relatively small. Moreover, the results on criterion validity rely on a cross-sectional basis (outcome at x+1 was associated with the ACT-SQ at x+1) and future studies including session-to-session ACT-SQ and outcome assessments should investigate whether the ACT-SQ at session x-1 predicts the outcome at session x (Rubel, Rosenbaum, & Lutz, 2017).

## General Discussion

A brief session questionnaire ACT-SQ was designed to obtain patient ratings on the in-session realization of the ACT components of psychological flexibility. The ACT-SQ was evaluated in ACT as well as CBT.

Results showed a one-factor solution (Research Question 1) and a good reliability (Research Question 2). All KMO values were good (.7 - .8) or great (.8 - .9) according to Hutcheson and Sofroniou (1999) or Field (2009). Moreover, all Bartlett’s tests were significant indicating that factor analysis was appropriate (Field, 2009). The loadings of all items were well above .45 as recommended in the literature (see for example, Bühner, 2010) and there were no cross-loadings. The one extracted factor could stand for the degree the in-session processes helped to increase the patient’s psychological flexibility. To further evaluate this hypothesis, a study is necessary investigating whether higher ACT-SQ session scores result in more improvements on established instruments measuring skills of psychologically flexibility (e. g., Acceptance and Action Questionnaire II; Bond et al., 2011).

Besides factor structure and reliability, we tested the convergent validity. Convergent validity was evaluated by correlating the ACT-SQ with the general change mechanisms proposed by Grawe (1997) since these mechanisms are considered to be relevant in all psychotherapies and because ACT processes might also be general change mechanisms as they mediated the outcome not only in ACT but also in CBT and multidisciplinary treatments (e. g., Åkerblom et al., 2015; Arch et al., 2012b). These analyses related to Research Question 3 revealed that the ACT-SQ is significantly associated with general change mechanisms (except for problem actuation in Study 1) according to Grawe (1997), most strongly with resource activation and mastery. A cautious clinical interpretation of these findings could be as follows: The content of the ACT-SQ items are associated with coping and self-efficacy as is the content of the items of the SACiP resource activation and mastery scales (Mander et al., 2013). Furthermore, the SACiP emotional bond and agreement on collaboration scales reflect the interaction processes between patient and therapist. The ACT-SQ items do not directly target this therapeutic relationship aspect. Hence, stronger associations of ACT-SQ and resource activation and mastery than with the alliance scales seem plausible. In summary, it is important to note that the ACT-SQ items are most strongly related to proximal items (resources and mastery) but also to items with more distanced but clinically relevant content (therapeutic alliance). This further underlines the validity of the measure. With regard to similarities and differences between ACT and CBT (Arch & Craske, 2008; Harley, 2015), most psychometric values were comparable between ACT and CBT, only a few differences emerged in the context of convergent validity (Research Question 4): associations between the ACT-SQ and problem actuation reached significance only in CBT. This could indicate more overlap between problem actuation and the ACT components in CBT than in ACT but it could also be related to the fact that the sample size of Study 1 (ACT) was not as large as the sample size of Study 2 (CBT). The same reasons might explain why the association between the ACT-SQ and the emotional bond was not significant anymore after controlling for multiple testing in Study 1 (ACT) but not in Study 2 (CBT).

In another step, we tested the criterion validity. This was related to Research Question 5 and the results showed significant associations between the ACT-SQ and outcome measures. It should be kept in mind, however, that relations with treatment outcomes were investigated only in CBT. Future research is necessary to evaluate whether the associations between the ACT-SQ and treatment outcomes are comparable or different between CBT and ACT. Finally, the factor structure, reliability, convergent validity, and criterion validity were comparable between earlier and later treatment phases (Research Question 6). Although differences in treatment phases have been highlighted (Ilardi & Craighead, 1994; Lambert, 2005), these differences did apparently no affect the psychometric values of the ACT-SQ.

A limitation of the current work is that only a patient version of the ACT-SQ was created and evaluated. A therapist version of the ACT-SQ would be an important tool that could be developed by future studies to get a more comprehensive picture of the therapeutic process. Other shortcomings of the studies at hand are that criterion validity was tested only in CBT but not in ACT. Moreover, contrasting the psychometric values in earlier vs. later treatment phases was possible only in CBT but not in ACT. Future studies on ACT are important to investigate criterion validity and similarities/differences between earlier and later ACT phases. A further limitation is that the mean of sessions attended was relatively high so that it remains unclear how well the results can be generalized to shorter psychotherapies. Moreover, we did not include other measures of ACT processes to correlate them with the ACT-SQ. Further validation studies should, therefore, compare ACT-SQ patient ratings with observer-based DUTARS ratings, since patient ratings are only one data source to rate in-session processes. Related to the factor analysis, setting the Kaiser criterion for determining the amount of factors at 1 is rather an arbitrary rule of thumb and an empirically founded way of determining the factors (i.e. Horn’s parallel analysis or Velicer’s MAP test) would have been a better method. In replication studies with larger samples, the factor structure needs to be tested with confirmatory factor analysis whether the instrument shows adequate model fit (Bühner, 2010). It is per se more probable for such a short questionnaire like the ACT-SQ to have a one-factor solution. Another suggestion for future research would be to enter additional predictors to the regression analyses to test interactions between patient characteristics (e. g., amount of diagnoses) and the impact the ACT-SQ has on the outcome. It would also be very interesting for future research to examine whether the factor structure of the ACT-SQ remains stable when patients are treated by specific ACT modules (open vs. engaged, see Villatte et al., 2016). The ACT-SQ might also be useful to measure adherence to ACT and to continuously track the ACT processes during psychotherapy. Parallel session-to-session assessments of the ACT processes and outcomes would allow investigating how the ACT processes are associated with patient progress on a between- and within-person level (Rubel et al., 2017). Such a systematic monitoring would also enable evaluating the ACT processes before and after sudden losses or sudden gains (Wucherpfennig, Rubel, Hofmann, & Lutz, 2017). Future research on group psychotherapy could also explore associations between group factors (see for example, Tasca et al., 2016, and Vogel, Blanck, Bents, & Mander, 2016) and ACT components.

In summary, the ACT-SQ has a clear factor structure, good reliability, shows strong associations to other validated psychotherapeutic change processes and is associated with treatment outcomes. Implications of this study are that the license-free ACT-SQ is a reliable and valid measure that can be used to measure how patients experience the in-session realization of ACT components.

## Supplementary Materials

The Supplementary Materials contain the English and German version of the ACT-SQ (for unrestricted access see Index of Supplementary Materials below).

10.23668/psycharchives.3462 Supplement 1 Supplementary materials to "Development and initial validation of a brief questionnaire on the patients’ view of the in-session realization of the six core components of Acceptance and Commitment Therapy

ProbstT.
MühlbergerA.
KühnerJ.
EifertG. H.
PiehC.
HackbarthT.
ManderJ.
 (2020). Supplementary materials to "Development and initial validation of a brief questionnaire on the patients’ view of the in-session realization of the six core components of Acceptance and Commitment Therapy"
[Questionnaire; English and German version]. PsychOpen. 10.23668/psycharchives.3462PMC964547836398148
