# Supplementary material for: Development and Initial Validation of a Brief Questionnaire on the Patients’ View of the In-Session Realization of the Six Core Components of Acceptance and Commitment Therapy
Source: Clin Psychol Eur. 2020 Sep 30;2(3):e3115. doi: 10.32872/cpe.v2i3.3115 (PMC9645478; doi:10.32872/cpe.v2i3.3115)
Supplement: Supplement 1 [file cpe-02-3115-s1.pdf]

Appendix A: English version of the ACT-SQ.

**The last (XY) psychotherapy session(s) helped me…**	does not apply	applies slightly	applies fairly well	applies to a great extent	applies extrem-ely
… to accept unpleasant feelings, thoughts or body sensations rather than fight them.	0	1	2	3	4
… to gain more inner distance from unpleasant feelings, thoughts or body sensations and to observe them rather than getting caught up in them.	0	1	2	3	4
… to stay in the here and now (in the present moment) rather than concerning myself with my future and my past.	0	1	2	3	4
… to realize that my feelings, thoughts and body sensations are part of me, but that I am more than my feelings, thoughts and body sensations.	0	1	2	3	4
… to recognize what is important to me in my life and what gives orientation to my life.	0	1	2	3	4
… to act in daily life according to what is important to me in my life and what gives orientation to my life.	0	1	2	3	4

Appendix B: German version of the ACT-SQ.

**Die letzte(n) (XY) Therapiesitzung(en) hat (haben) mir geholfen …**	trifft nicht zu	trifft kaum zu	trifft ziemlich zu	trifft deutlich zu	trifft extrem zu
… unangenehme Gefühle, Gedanken oder Körperempfindungen mehr zuzulassen anstatt mich gegen sie zu wehren.	0	1	2	3	4
… mehr innere Distanz zu unangenehmen Gefühlen, Gedanken oder Körperempfindungen aufzubauen und sie zu beobachten anstatt mich von ihnen vereinnahmen zu lassen.	0	1	2	3	4
… im Hier und Jetzt (in der Gegenwart) zu bleiben anstatt mich mit der Zukunft und meiner Vergangenheit zu beschäftigen.	0	1	2	3	4
… zu erkennen, dass meine Gefühle, Gedanken und Körperempfindungen zwar Teile von mir sind, aber dass ich mehr bin als meine Gefühle, Gedanken und Körperempfindungen.	0	1	2	3	4
… zu sehen, was mir im Leben wichtig ist und meinem Leben Orientierung verleiht.	0	1	2	3	4
… im Alltag umzusetzen, was mir im Leben wichtig ist und meinem Leben Orientierung verleiht.	0	1	2	3	4
